# Extrinsic electric field modulates neuronal development and increases photoreceptor population in retinal organoids

**DOI:** 10.3389/fnins.2024.1438903

**Published:** 2024-11-29

**Authors:** Deepthi S. Rajendran Nair, Anika Gupta, Ege Iseri, Tianyuan Wei, Le Tam Phuong Quach, Magdalene J. Seiler, Gianluca Lazzi, Biju B. Thomas

**Affiliations:** ^1^Department of Ophthalmology, USC Roski Eye Institute, University of Southern California, Los Angeles, CA, United States; ^2^Department of Biomedical Engineering, Viterbi School of Engineering, University of Southern California, Los Angeles, CA, United States; ^3^Departments of Physical Medicine and Rehabilitation; Ophthalmology; Anatomy and Neurobiology, University of California, Irvine, Irvine, CA, United States; ^4^Stem Cell Research Center, University of California, Irvine, Irvine, CA, United States; ^5^Department of Electrical and Computer Engineering, Viterbi School of Engineering, University of Southern California, Los Angeles, CA, United States; ^6^USC Ginsburg Institute for Biomedical Therapeutics, University of Southern California, Los Angeles, CA, United States

**Keywords:** retinal organoids, electrical stimulation, electrical field, photoreceptors, retinal degeneration, stem cell differentiation

## Abstract

**Introduction:**

Considering the significant role played by both intrinsic and extrinsic electric fields in the growth and maturation of the central nervous system, the impact of short exposure to external electric fields on the development and differentiation of retinal organoids was investigated.

**Methods:**

Retinal organoids derived from human embryonic stem cells were used at day 80, a key stage in their differentiation. A single 60-minute exposure to a biphasic electrical field was administered to assess its influence on retinal cell populations and maturation markers. Immunohistochemistry, qPCR, and RNA sequencing were employed to evaluate cell type development and gene expression changes.

**Results:**

Electrical stimulation significantly enhanced neuronal development and increased the population of photoreceptors within the organoids. RNA sequencing data showed upregulated expression of genes related to rod photoreceptors, Müller cells, horizontal cells, and amacrine cells, while genes associated with retinal pigment epithelium and retinal ganglion cells were downregulated. Variations in development and maturation were observed depending on the specific parameters of the applied electric field.

**Discussion:**

These findings highlight the significant impact of extrinsic electrical fields on early retinal development and suggest that optimizing electrical field parameters could effectively address certain limitations in retinal organoid technology, potentially reducing the reliance on chemicals and small molecules.

## Introduction

Retinal organoids (ROs) recapitulate the spatial and temporal progression of *in vivo* human retinal development. Cowan et al. (2020) found that the gene expression and cell types of 38-week-old ROs closely resemble the newborn human retina. While ROs may imitate certain stages of retinogenesis associated with the development of the neural retina, they do not encompass the entirety of the ocular system and thus lack full representation of the whole human eye (Nakano et al., 2012). Furthermore, the retinal cells in ROs exhibit a stratified structure and possess cell–cell connections akin to those observed in the *in vivo* retina (Grigoryan, 2022).

The ROs are generated from pluripotent stem cells by treating them with various cocktails of small molecules having composition designed to mimic the cues received by cells throughout *in vivo* retinal development (Bell et al., 2020). They initially develop into the optic vesicle (OV) after embryoid body (EB) formation (Khan et al., 2016). OVs then undergo invagination, resulting in the creation of the optic cup, with the inner layer becoming the neural retina (NR) and the outer layer becoming the retinal pigmented epithelium (RPE), respectively (Capowski et al., 2016). The neural retinal structures are subsequently isolated and cultured in suspension as retinal organoids (ROs), with the ROs initially developing retinal ganglion cells (RGCs). Later they become stratified containing more photoreceptors, bipolar cells, RGCs, horizontal cells, amacrine cells, and Müller cells that are aligned in multiple layers (Nakano et al., 2012; Afanasyeva et al., 2021).

As in the case of early eye development, during RO development, the neural retina is formed by ventral progenitors, which later give rise to all retinal neurons and glial cells. MiTF, FGF1, FGF11, FGF9, FGF13, TGFβ, Notch, retinoids, and Gas1 are responsible for the diversification and stabilization of the two major visual domains (RPE and NR) in the eye development (Gamm et al., 2019; Falix et al, 2012). Among these, Notch signaling pathway activation is necessary for proper retinal development but not for neuronal differentiation (Mills and Goldman, 2017). Starting around day 80, early progenitors of cones and rods begin to appear in ROs (Afanasyeva et al., 2021). They develop over months, reaching a maximal *in vitro* maturation state around the age of 30 weeks with current methods (Cowan et al., 2020).

The different cell types in retinal organoids (ROs) has unique properties. They are similar in composition and function to the native retina, can self-organize, and allow multiple cell types to communicate with each other. These advantages make organoids ideal for accurate human disorder models and diagnostics. Retinal organoids (ROs) are considered a promising source for photoreceptor replacement therapies and also enable the isolation of retinal ganglion cells (RGCs) and Müller cells using cell surface markers for broader therapeutic applications (Grigoryan, 2022). Our laboratory has conducted transplantation experiments using RO sheets in rat disease models showing survival and maturation of transplanted photoreceptors and signs of visual improvements (Lin et al., 2020; Nair et al., 2021).

Following the pioneering work by Nakano et al. (2012) and Meyer et al. (2011), numerous protocols have been developed for generating retinal organoids from human induced pluripotent stem cells (hPSCs) (Bell et al., 2020). Investigators have employed techniques using signaling factors/small molecules at various time points and concentrations to augment the RO yield and increase the PR population. Conditions have since been developed that promote differentiation of retinal organoids with a more stereotypical complement and robust population of photoreceptors (PRs), capable of maturing several 100 days in culture (Bell et al., 2020). Modified culture techniques have been used to accelerate differentiation in early developmental cell populations (Wahlin et al., 2017; DiStefano et al., 2018; Zerti et al., 2020, 2021), to increase the number of PRs (Mellough et al., 2015; Luo et al., 2018) to help earlier development of outer segments (Ovando-Roche et al., 2018), to improve the ratio of cone-to-rod PRs (Kim et al., 2019), and to enhance RGC migration and maturation (Gao et al., 2016).

Given its current importance, advancing RO technology is crucial by ensuring uniformity among ROs within the same culture dish and optimizing them to enrich for desired cell types, facilitating their use in transplantation experiments and disease modeling. The impact of external electrical fields (EFs) on central nervous system (CNS) development and maturation has been extensively studied using *in vivo* and *in vitro* models. It is well known that external electrical and electromagnetic fields can influence the development and maturation of CNS (Fröhlich, 2014; Thompson et al., 2014; Kaplan et al., 2016; Feng et al., 2017; George et al., 2017; O’Hara-Wright et al., 2022). In a recent optic nerve injury model, transcorneal electrical stimulation was shown to improve the morphology and survival of retinal ganglion cells (RGCs) (Stewart et al., 2015). In another study electrical currents delivered to the eye were investigated in rat models of retinitis pigmentosa (Hanif et al., 2016; Alekseichuk et al., 2019) showing improved neuronal preservation and visual functional benefits presumably by influencing pathways associated with neuronal protection and apoptosis. Recently, we demonstrated the benefits of extraocular electrical stimulation in activating the retinal neural circuitries *in vivo*, leading to improved vision in retinal degenerate RCS rats (Calle et al., 2023).

The above investigations suggest the possibility of modulating the development and maturation of ROs through the application of extrinsic electrical fields. Previously, *in vitro* studies have demonstrated the influence of electrical field (EF) on stem cell differentiation and maturation into organoids (Yu et al., 2022). Electrical stimulation-induced human neural stem cells to *β*-III Tubulin (Tuj1) expressing neurons with clusters of neurons exhibiting longer neurites and greater branching than unstimulated cultures (Phillips et al., 2014). Electrical stimulation of human neural progenitor cells alters their transcriptome including changes to the VEGF-A pathway and genes involved in cell survival, inflammatory response, and synaptic remodeling (Henrich-Noack et al., 2013). Studies conducted by Kondo et al. demonstrated that electrical stimulation promotes the differentiation of embryonic stem cells into a diverse range of neuronal cell types, whereas growth factor-induced ES cells tend to differentiate into more limited neuronal cell types (Yamada et al., 2007). Recent studies using isolated rat RGCs demonstrated that a new class of asymmetric, charge-balanced waveforms effectively direct RGC axon growth *in vitro* without compromising cell viability (Gokoffski and Zhao, 2019; Peng et al., 2023). These findings suggest the possibility of modulating the development and maturation of ROs through the application of extrinsic EFs.

The strength of the Asymmetric Charge Balanced (ACB) waveform lies in its ability to combine the safety of the traditional biphasic waveform with the efficacy of direct current (DC). This is achieved through a longer working phase and a shorter charge-balancing phase. A significant finding from the above study was that phase width ratio of 1:4 between the charge-balancing and working phases resulted in the highest migration rate of axons toward the target direction. Increasing the asymmetry beyond this ratio had no effect on the migration rate. To achieve a complete charge balance with a phase width ratio of 1:4, an amplitude ratio of 4:1 between the two phases was necessary. Another important outcome was the determination of the threshold stimulation amplitude for promoting electrotaxis, which was approximately 1 V/cm. While higher amplitudes increased the migration ratio toward the target, saturation occurred at 2 V/cm, which is the maximum ratio achieved using DC.

In this new investigation using extrinsic EF, we aimed to modulate the RO development with emphasis on photoreceptor maturation. Based on the findings from our previous studies we selected two ACB waveforms, utilizing a phase width ratio of 1 ms:4 ms and an amplitude ratio of −4 V:1 V or − 8 V:2 V for the cathodic and anodic phase, respectively. The EF parameters chosen were aimed to maximize the efficacy of the applied EFs while maintaining safety for the ROs. In the present study, only D80 ROs were used. This is due to the specific developmental timeline during which a diverse array of neuron subpopulations, including RGCs, interneurons, and PRs, start to coexist.

## Materials and methods

### Culture of hESC-derived retinal organoids (RO)

NIH-registered H9 human embryonic stem cells (hESCs), genetically modified with a green fluorescent protein (GFP) tagged to the CRX gene (CRX-GFP ESCs) obtained at USC through a Material transfer agreement (MTA) with the University of Newcastle (provided by Dr. Seiler lab University of California, Irvine) was used for making the ROs. ROs were generated using a protocol previously described with minor modifications (Zhong et al., 2014; Xue et al., 2021). Briefly, CRX-GFP H9 cells were cultured in mTeSR 1 media (STEMCELL Technologies, Vancouver, BC, Canada) and maintained at 37°C in a humidified 5% CO_2_ incubator (Nuaire, Plymouth, MN, USA). Passaging was performed at 80% confluency using ReLeSR (STEMCELL Technologies, Vancouver, BC, Canada). Cell expansion was carried out on BD GFR Matrigel-coated plates. For the differentiation of ROs, Accutase (Nacalai Inc., Kyoto, Japan) was introduced to the confluent stem cell culture to generate a single-cell suspension. Subsequently, the cells were transferred to an 800-μm micro-well EZSPHERE 6-well plate (Nacalai U.S.A., Inc., San Diego, CA, USA) and centrifuged at 100 g for 3 min using a plate centrifuge, initiating the formation of embryoid bodies (EB) from day 1 to 7 in the EZSPHERE microwells by gradually replacing neuronal induction medium. On day 8, EBs were seeded onto 1% growth factor-reduced Matrigel (Corning, NY, USA) coated culture dishes. Neural Induction Media (NIM) Dulbecco’s modified eagle medium (DMEM)/F12 (1:1) (Gibco, Waltham, MA, USA), 1% N2 supplement (Gibco), 1x minimum essential media non-essential amino acids (NEAA) (Gibco), 1x L-glutamine (Gibco), and 2 μg/mL heparin (Sigma-Aldrich, St. Louis, MO, USA) were used from day 8 onwards, with media changes every 2 days. Embryoid bodies attached to and spread across the culture dish, initiating differentiation into eye field structures. From day 19 to 41, the media transitioned to NIM containing DMEM/F12 (1:1) supplemented with 2% B27 supplement (50X) (minus vitamin A, Gibco), 1x NEAA, 1x L-glutamine, and 2 mg/mL heparin (Sigma, Burlington, USA). Between days 40–50, retinal eye fields were carefully cut out from the culture dish and transferred to ultra-low attachment 24-well plates (Corning, NY, USA). From day 19 to 41, the media transitioned to NIM containing DMEM/F12 (1:1) supplemented with 2% B27 supplement (50X) (minus vitamin A, Gibco, MT, USA), 1x NEAA, 1x L-glutamine, and 2 mg/mL heparin. Starting day 42, the organoids were cultured with media containing DMEM/F12 (1:1) supplemented with 2% B27 Plus Supplement (50X) (Gibco, MT, USA), 1x NEAA, 1x L-glutamine, 2 ug/ml heparin, 100 μM taurine (Sigma, Burlington, USA), and 10% fetal bovine serum (FBS; Gibco, Montana, USA). ROs were selected that contained an outer transparent layer and had developed a hollow spherical shape with a laminated structure, as observed under phase contrast and dissection microscope (McLelland et al., 2018; Thomas et al., 2021). All possible efforts were taken to minimize variability between experiments based on RO shape and size. For this, a strict selection criterion based on microscopic evaluation of the ROs was followed. ROs of different shape and sizes were equally distributed across the different experimental groups.

### RO stimulation using electrical field (EF)

**Figure 1:**
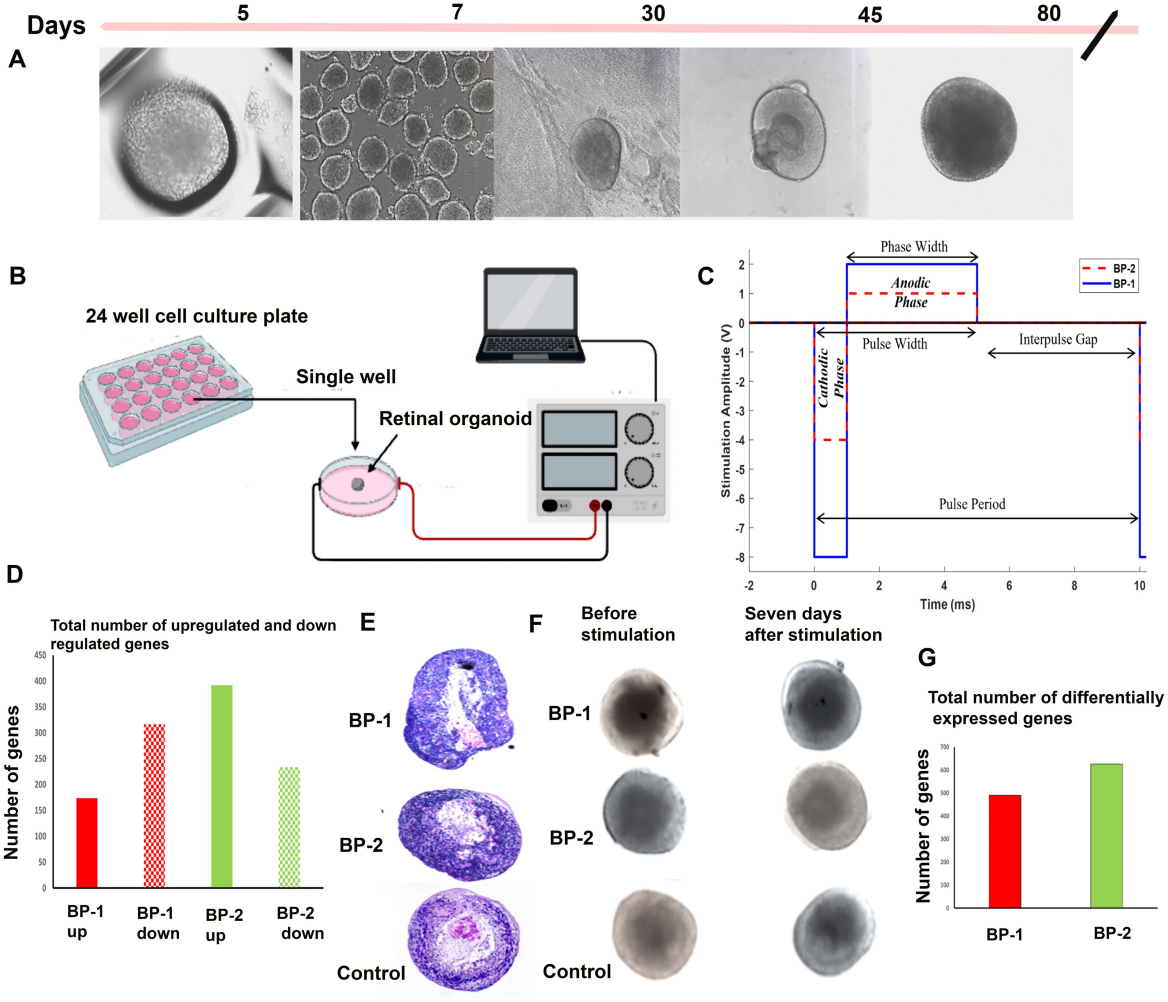
Electrical stimulation of D80 retinal organoids (ROs) followed by 7 days in culture. Diagrammatic representation of the study summary. (A) Different RO developmental stages, (B) electrical stimulation setup used (BP-1 and BP-2), (C) waveforms used for BP-1(blue continuous line) and BP-2 (red dotted line). (D) RNA sequencing- total number of differentially expressed genes in two different electrical stimulation setups, (E) hematoxylin and Eosin (H&E) images of organoids after stimulation (D87), (F) phase contrast images of organoids after stimulation (D87). (G) RNA sequencing- total number of upregulated and downregulated genes in two different electrical stimulation setups.

ROs that contain multiple neuronal cell types, including photoreceptors (PRs), retinal ganglion cells (RGCs), intermediate neurons, and Müller cells, can be influenced by external electrical field (EF). We devised a new method to grow ROs in culture with concurrent EF stimulation to promote photoreceptor differentiation. The EF parameters were chosen based on the *in vitro* electrical stimulation studies conducted using RGCs (Gokoffski and Zhao, 2019; Peng et al., 2023) that are both effective and safe. Day 80 (Figure 1A) ROs were placed inside 24 well tissue culture plates. Pre-cut Linbro plate sealer was placed over the 24 well plates and attached to the chamber walls, serving as the roof of the chamber. The dimensions of the chamber through which the current was passed measured 16 mm (diameter) and 20 mm (height). Platinum (Pt) wire electrodes (0.25 mm diameter; P1 Technologies, Boerne, TX), each 10 mm long, were placed at either end of the circular chamber, separated by the chamber’s diameter. Because the electrodes made direct contact with the media in which the ROs were placed, platinum (Pt) was chosen as a biocompatible material to minimize toxicity (Dymond et al., 1970; Stensaas and Stensaas, 1978). Under proper sterile conditions, the ROs were exposed to 1-h EF stimulation under one of the following three EF conditions: (1) BP-1 (− 8 V for 1 millisecond, 2 V for 4 milliseconds, 0 volts for 5 milli seconds), (2) BP-2 (−4 volt for 1 millisecond, 1 volt for 4 milliseconds, 0 volts for 5 milli second), and (3) control (no electrical stimulation sham). While the amplitudes exceed the water window of Pt microelectrodes (Cogan, 2008), the biphasic nature of the waveform, along with the short phase widths, ensures that conduction is primarily capacitive and that toxic charge injection via faradaic conduction is minimized (Merrill and Stecker, 2022). EF was applied using an Arbitrary Waveform Generator (RIGOL DG 822 2-Channel AWG, Portland, OR, 97223). Two tungsten needle electrodes were placed at either side of the organoid, 5 mm apart, to measure the voltage gradient across the organoid. These values were recorded in MATLAB (MathWorks, Natick, MA) using a Keysight DSOX2014A oscilloscope. To ensure that our biphasic waveforms were charge-balanced, total injected charge (area under the plot, Figure 1C) was monitored throughout the experiment. The total charge of the cathodic phase was divided by that of the anodic phase to give a charge balance ratio. A voltage-controlled stimulation protocol ensured that the charge transfer process was capacitor-coupled and charge balance could be maintained as the working electrode discharges by shorting with the counter electrode during the interpulse interval (Merrill, 2010). After stimulation, the ROs were cultured for 7 days before subjected to various morphological assessments and gene expression assays (Figures 1D–G).

Microscopic evaluation was conducted to select the ROs having uniform shape and size for further analysis. Using a phase contrast microscope, the organoids were screened based on the presence of outer transparent layer with a hollow spherical shape and a laminated structure. All the ROs were divided into 3 groups of equal numbers based on the experimental condition (BP-1, BP-2 and Control). After the initial screening, 30 ROs from 3 different batches were selected for histological studies, and 135 ROs were used for molecular assays (*n* = 3, triplicates of 15 in each group).

### Phase contrast imaging and histological examination

Phase-contrast imaging of the ROs before and after the EF exposure was conducted under 10X magnification. For detailed histological examination, ROs were fixed in 4% paraformaldehyde for 30 min at room temperature. Sections that were cut using a microtome (5 μm) were deparaffinized prior to hematoxylin and eosin (H&E) staining. H&E images were used for gross morphological assessments.

### Immunostaining

Paraffin-embedded sections (5 μm) of the ROs were permeabilized with 0.5% Triton X-100 (Catalog No.PI85111, Thermo Scientific™, Waltham, Massachusetts) for 20 min and blocked for 1 h at room temperature with 1% BSA and 0.5% Triton X-100 in PBS. Then, sections were incubated in blocking buffer for 12 h at 4°C with primary antibodies. After this, the sections were washed and incubated with secondary antibody for 1 h. The primary and secondary antibodies used in the study are given in Supplementary Table S2. Sections were stained for 10 min with DAPI (Catalog No.ab228549, abcam, USA) before imaging.

### Quantitative polymerase chain reaction (qPCR)

Reverse Transcription: cDNA was generated from the extracted RNA template using Invitrogen SuperScript IV First-Strand Synthesis System. An RNA-negative control and RNA-positive control with the absence of Reverse Transcriptase was used in RT and run in parallel with experimental samples. qPCR was performed per Azenta Life Sciences (South Plainfield, NJ, USA) SOP using PCR probes listed in Supplementary Table S1. The samples were processed in technical replicates of 3 per target. Data analysis was performed by Azenta Life Sciences using QuantStudio™ Real-Time PCR Software.

### Image quantification

Quantification of the recoverin, CRX and vimentin-positive cells were done using ImageJ software.^1^ The number of positive stained cells for each marker was analyzed and normalized to the number of DAPI labeled cells in a given region. Up to 12 regions (1cm^2^) were analyzed per organoid, and 6 organoids were analyzed per condition. The result was further analyzed using Microsoft Excel and Graph-Pad Prism 8.2.1.

### RNA sequencing

RNA extraction, library preparation, sequencing, and analysis were conducted at Azenta Life Sciences (South Plainfield, NJ, USA) as follows: Total RNA was extracted from fresh frozen cell pellet samples using Qiagen RNeasy Plus Universal mini kit following manufacturer’s instructions (Qiagen, Hilden, Germany). Library Preparation with PolyA selection and Illumina Sequencing.

RNA samples were quantified using Qubit 2.0 Fluorometer (Life Technologies, Carlsbad, CA, USA), and RNA integrity was checked using Agilent TapeStation 4,200 (Agilent Technologies, Palo Alto, CA, USA). RNA sequencing libraries were prepared using the NEBNext Ultra RNA Library Prep Kit for Illumina using the manufacturer’s instructions (NEB, Ipswich, MA, USA). Briefly, mRNAs were initially enriched with Oligod (T) beads. Enriched mRNAs were fragmented for 15 min at 94°C. First-strand and second-strand cDNA were subsequently synthesized. cDNA fragments were end-repaired and adenylated at 3’ends, and universal adapters were ligated to cDNA fragments, followed by index addition and library enrichment by PCR with limited cycles. The sequencing library was validated on the Agilent TapeStation (Agilent Technologies, Palo Alto, CA, USA), and quantified by using Qubit 2.0 Fluorometer (Invitrogen, Carlsbad, CA) as well as by quantitative PCR (KAPA Biosystems, Wilmington, MA, USA). The sequencing libraries were clustered on a flowcell. After clustering, the flowcell was loaded on the Illumina instrument (4,000 or equivalent) according to the manufacturer’s instructions. The samples were sequenced using a 2x150bp Paired-End (PE) configuration. Image analysis and base calling were conducted by the Control software. Raw sequence data (.bcl files) generated the sequencer were converted into fastq files and de-multiplexed using Illumina’s bcl2fastq 2.17 software. One mismatch was allowed for index sequence identification. After investigating the quality of the raw data, sequence reads were trimmed to remove possible adapter sequences and nucleotides with poor quality. The trimmed reads were mapped to the reference genome available on ENSEMBL using the STAR aligner v.2.5.2b. The STAR aligner is a splice aligner that detects splice junctions and incorporates them to help align the entire read sequences. BAM files were generated because of this step. Unique gene hit counts were calculated by using feature counts from the subread package v.1.5.2. Only unique reads that fell within exon regions were counted.

After the extraction of gene hit counts, the gene hit count table was used for downstream differential expression analysis. Using DESeq2, a comparison of gene expression between the groups of samples was performed. The Wald test was used to generate *p*-values and Log2 fold changes. Genes with adjusted p-values <0.05 and absolute log2 fold changes >1 were called as differentially expressed genes for each comparison. A Gene Ontology (GO) analysis was performed on the statistically significant set of genes by implementing the software GeneSCF. The mgi GO list was used to cluster the set of genes based on their biological process and determine their statistical significance. A PCA analysis was performed using the “plotPCA” function within the DESeq2 R package. Plot that shows the samples in a 2D plane spanned by their first two principal components were created. The top 500 genes, selected by highest row variance, were used to generate the plot.

## Results

### Acceleration of RO development after electrical stimulation

**Figure 2:**
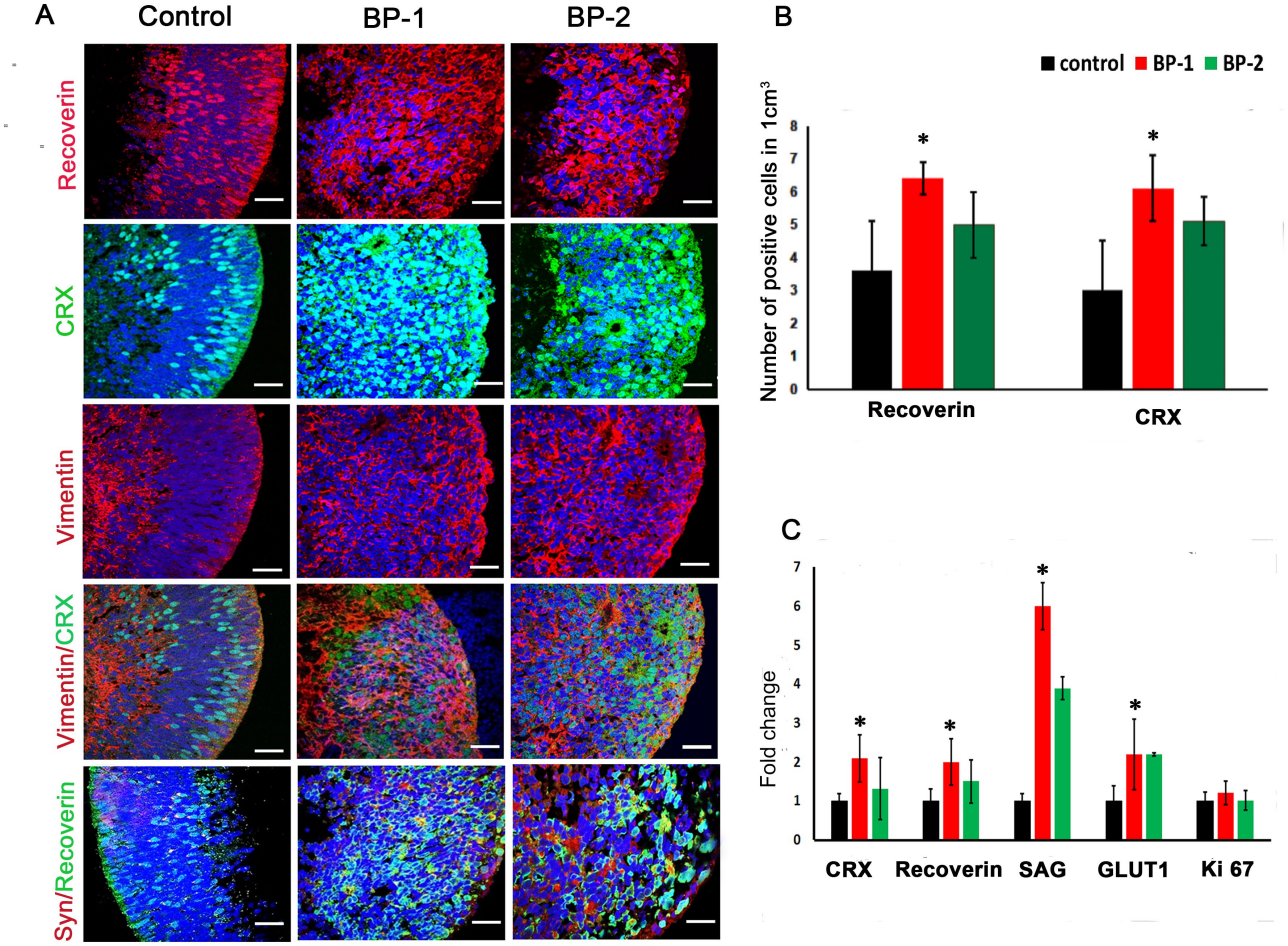
Immunostaining and qPCR of D87 retinal organoids that were subjected to 2 different levels of electric stimulation (BP-1 and BP-2) showing increased expression of major retinal cell types. (A) Confocal images after immunostaining of ROs 7 days after electric stimulation showing different retinal cell types such as recoverin CRX, vimentin and synaptophysin staining. Blue color, DAPI- nuclear stain. 60 X magnification, Scale bar 100 μm. (B) Total number of cells that expressed different photoreceptor markers in the control group (black) and electrically stimulated RO group (BP-1, red, BP-2, green). (C) qPCR analysis of gene expression in D87 retinal organoids in BP-1 and BP-2 electrical stimulation groups and control (non-stimulated) group. Photoreceptor markers (CRX, recoverin, SAG), synaptic marker (vGLUT1) after 1 h of electrical stimulation at D80 followed by 7 days in culture (D87). Gene expression was normalized to GAPDH and was compared to two different electrical stimulation setups. Error bars represent SEM.*p ≤ 0.05.

Microscopic evaluation of the ROs after stimulation was performed using phase contrast microscope (Figure 1F) suggested absence of apparent alteration in the RO structure after EF stimulation. H&E images suggested RO enrichment based on an increased concentration of cells exhibiting deeply stained nuclei in the core region of the RO (Figure 1E). Immunohistochemistry (Figure 2A) revealed substantial modification in the level of photoreceptor and Müller cell marker expressions in EF-stimulated ROs (EF ROs) compared to the unstimulated age-matched control ROs. EF ROs exhibited considerable increase in the expression of the general photoreceptor marker, recoverin. Recoverin is a calcium-binding protein present mainly in retinal rods, cones, and cone bipolar cells. In addition, increased expression of photoreceptor marker, cone-rod homeobox (CRX), and Müller cell marker vimentin was also noticed in EF ROs. CRX is a photoreceptor-specific transcription factor that plays a role in the differentiation of photoreceptor cells.

### EF-induced changes in the expression of key RO developmental genes

Quantitative polymerase chain reaction (qPCR) gene analysis was used to compare expression patterns between day 80 electrically stimulated ROs (EF ROs) and age-matched non-stimulated ROs (Figure 2C). There was a significant increase in the expression of general photoreceptor markers SAG, Recoverin, CRX, and synaptic marker vGLUT1. No significant cell proliferation was observed based on Ki 67 expression (Figure 2C).

### Differential expression of genes and pathways in electrically stimulated ROs (EF ROs)

**Figure 3:**
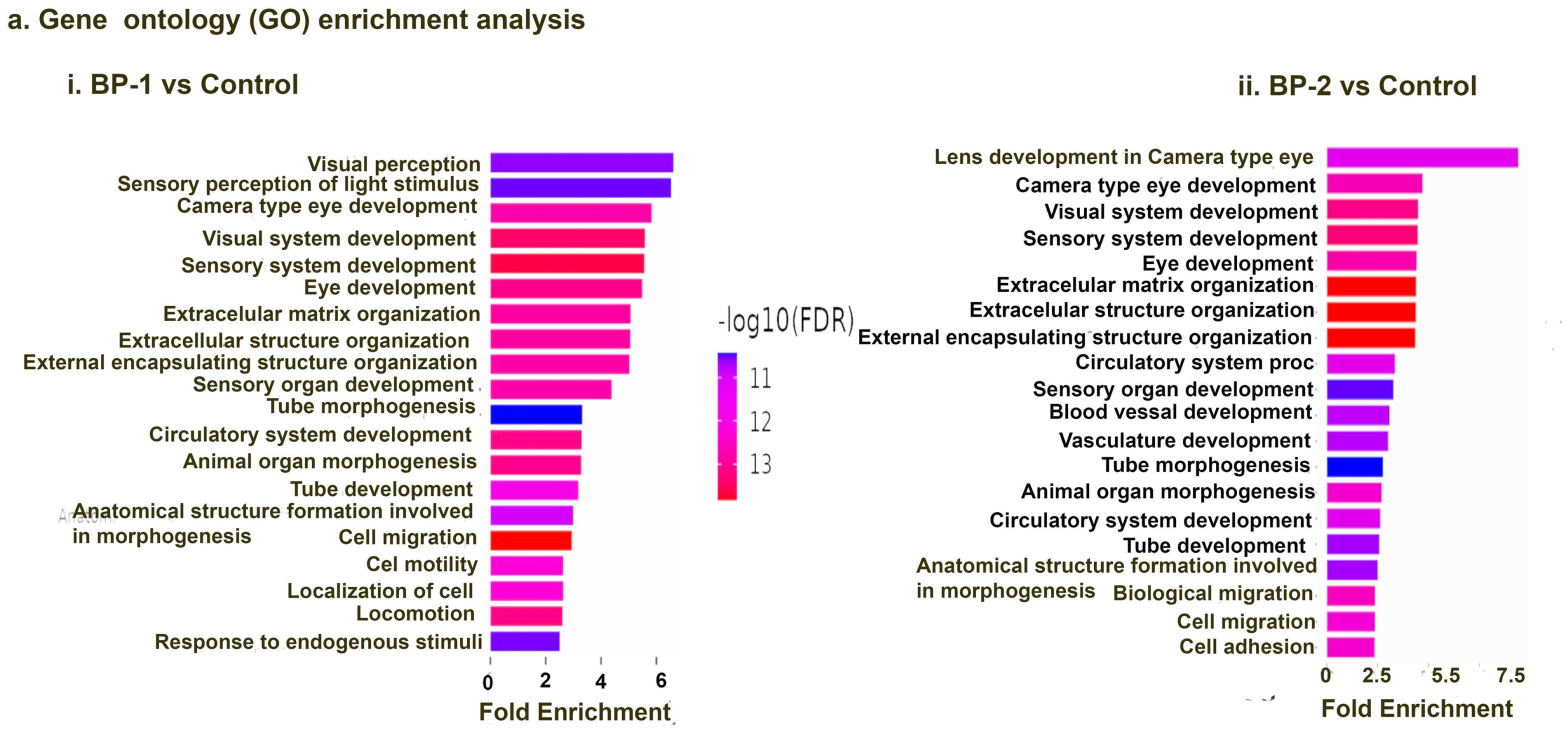
Gene ontology (GO) analysis data. Top 10 GO biological category annotations for the differentially expressed genes (DEGs) identified based on the RNA-seq data for control vs. electrically stimulated retinal organoid samples (BP-1 vs. control, BP-2 vs. control). Each GO group that is significantly overexpressed (*p* < 0.05) is included in this list.

**Figure 4:**
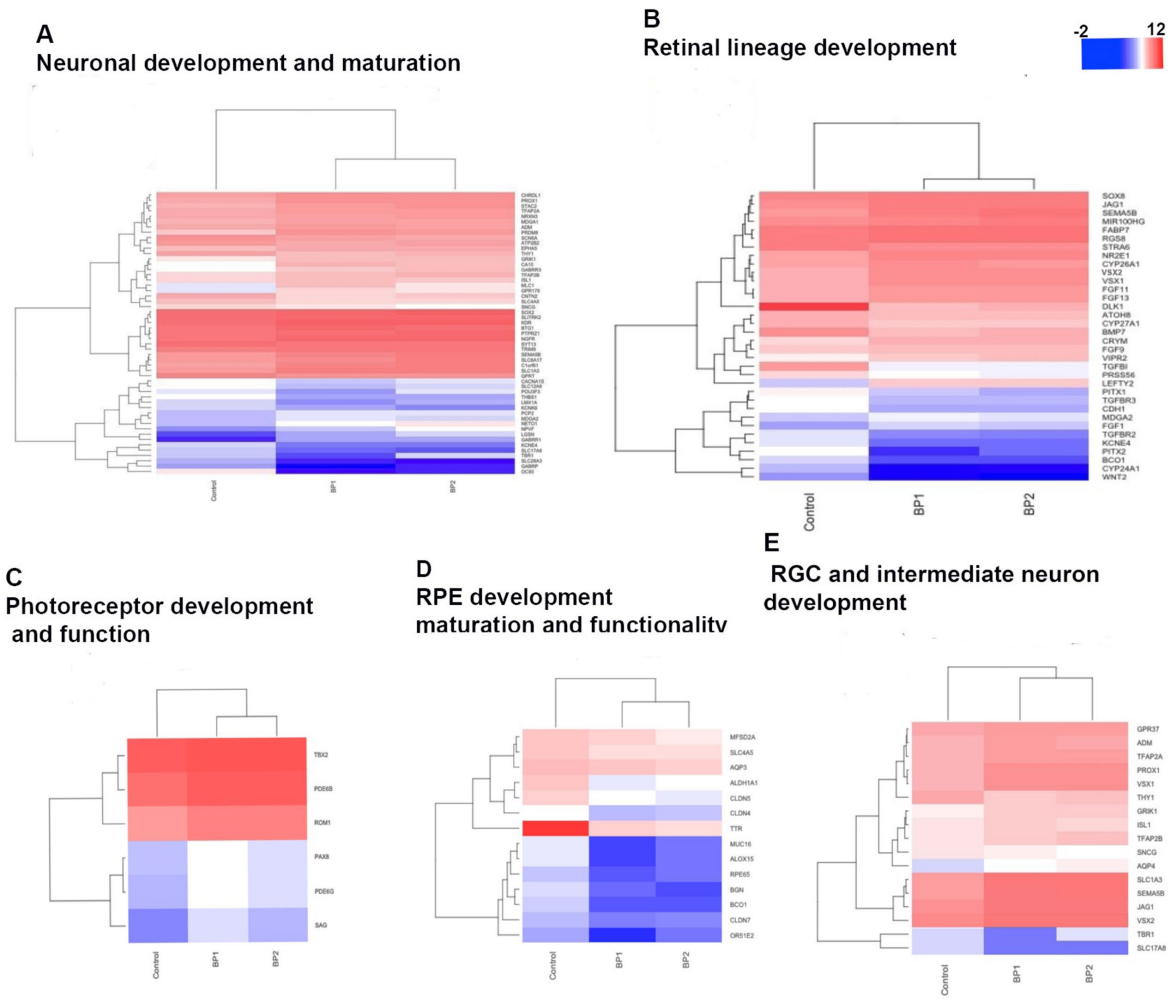
Ingenuity Pathway Analysis (IPA) data. IPA data of differentially expressed retinal organoid genes showed as a graphical summary representing networks of the major pathways identified as the most significant in the differential transcriptomics data (*p* < 0.05). (A) BP-1 vs Control, (B) BP2-vs Control.

RNA sequencing studies using EF-stimulated ROs (ROs exposed to EF followed by 7 days culture under normal conditions) were performed. Significant changes in the gene expression were estimated based on differential expression (DE) analysis. Initial DE analysis data indicated the presence of 492 DE genes in the BP-1 EF group and 603 DE genes in the BP-2 EF group (*p*-value <0.05 and absolute log2 fold change >1, Figures 1D,G). We employed three different analysis techniques to correlate the data based on DE genes with RO development and maturation. This included DESeq (Differential gene expression analysis based on the negative binomial distribution), IPA (Ingenuity Pathway Analysis), and GO (Gene Ontology) analysis. GO biological category analysis of DEGs showed that genes related to retinal development and function remained top on the list in both BP-1 and BP-2 groups (Figure 3). Interestingly, the highest level of differences was observed in genes related to visual perception; that was more apparent in BP-1 group (Figure 3). Differences between BP-1 and BP-2 groups were also noticed in the clusters of genes belonging to the various functional groups (Supplementary Figure S1). IPA investigated significantly involved pathways and cellular functions in ROs after EF (Figure 4, Supplementary Figure S2). In the BP-1 group, among the top in the list included the visual phototransduction pathway and various neuronal signaling pathways (Figure 4A, Supplementary Figure S2A). Ingenuity Pathway Analysis of the visual phototransduction pathway in BP-1 group is presented in Supplementary Figure S3. There was no expression of the above pathway in the BP-2 stimulation group (Figure 4B). This suggests major differences between the two EF paradigms in influencing the RO development and maturation (also see, Supplementary Figures S2 A,B).

**Figure 5:**
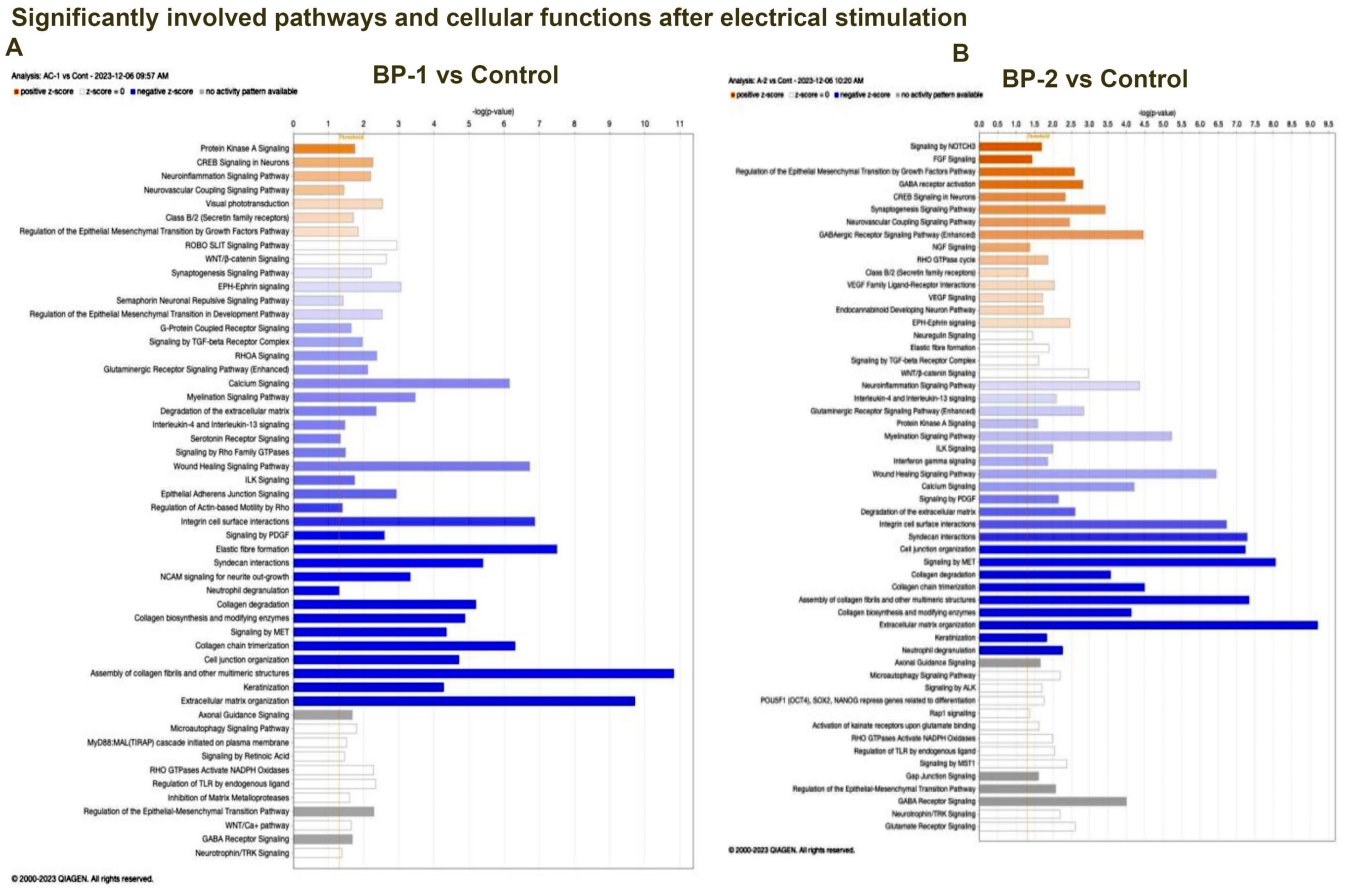
Heat map showing the differentially expressed genes representing the major retinal organoid developmental pathways. Transcriptional profiling was performed and heatmaps were generated to compare differentially expressed genes (DEGs) between BP-1, BP-2, and control retinal organoids at D87. (A) Neuronal development and maturation, (B) Retinal lineage development, (C) Photoreceptor development and function, (D) RPE development maturation and functionality, (E) RGC and Intermediate neuron development.

Based on DESeq analysis for differentially expressed genes, genes that are associated with early retinal development and maturation showed significant differences between BP-1 and BP-2 (Figure 5, Supplementary Figure S4, Supplementary Tables S3a-n). Interestingly several gene expression pathways that are involved in early eye development showed significant downregulation or remained unaffected (Figure 5). On the other hand, significant upregulation of key genes directly related to photoreceptor development and function was noticed (Figure 5C, Supplementary Tables S3e,f). Upregulation of genes related to photoreceptor development and function predominantly belonged to the rod pathway. Interestingly, this increase in expression was mostly limited to the BP-1 group (Figure 5B, Supplementary Tables S3e,f), suggesting a profound influence of stimulation parameters on retinal developmental pathways. Other major changes observed were in the downregulation of genes associated to RPE development and its maintenance (Figure 5D, Supplementary Tables S3m,n). While RGC genes showed downregulation, genes associated with the development and maturation of bipolar, horizontal, amacrine, and retinal ganglion cells were mostly upregulated (both in BP-1 and BP-2, Figure 5E). Other major changes associated with EF stimulation included an increase in the gene expression pattern of Müller cells (Figure 5E).

## Discussion

In mammals, retinal development involves several key steps: initially, the proper proportions of seven key retinal cell types are established, with ganglion cells being the only type regulated by cell death. Following this, cells migrate to their designated locations, neurons form synaptic connections, and synaptic refinement occurs to create the mature retinal circuitry. Notably, the development includes the emergence of RGCs first, followed by amacrine cells, Müller cells, bipolar cells, and horizontal cells, with photoreceptors finalizing the top layers. Additionally, during development, intermediate circuits are formed and restructured, generating spontaneous retinal waves through transient networks of electrical and chemical signaling. Also electric fields arise from ion channels and pumps, membrane potential differences, extracellular matrix and cell–cell interactions. Such endogenous electrical activities guide cell migration, polarity, and organization to ensure proper retinal formation and function (Ford and Feller, 1995). This suggests that extrinsic electrical fields can have a profound influence on mammalian retinal development.

Retinal organoids (ROs) are a unique *in vitro* model system due to their ability to self-organize into distinct layers that closely mimic the human retina’s structure and function. This includes the formation of photoreceptors, ganglion cells, bipolar cells, Müller glia, and other retinal cell types while also capable of exhibiting electrical signaling patterns similar to those *in vivo*, such as synaptic activity (Bell et al., 2020). In our study, 8 day old (D80) ROs were analyzed 7 days after a single 60-min exposure to a specific electrical stimulation paradigm. Exposure to the electrical field (EF) resulted in significant increase in the number of photoreceptors, Müller cells, and several non-photoreceptor retinal neural cell types along with improved synaptic activities. RNA sequencing (RNA-seq) data has proven to be highly informative in elucidating the impact of EF stimulation on diverse retinal cell populations, as well as the alterations observed in their developmental trajectory.

Although the transcription factors such as PAX6, SFRP2, CDH2, MAP2, RAX, SIX3, LHX2, SIX6, NR2F2, and OTX2 that regulate the early genesis of the eye (Mathers et al., 1997; Chow et al., 1999; Phillips et al., 2014; O’Hara-Wright and Gonzalez-Cordero, 2020) were not affected by EF, other critical pathways involved in retinal development and maturation were differentially expressed in ROs after exposed to the EF. Vsx2, FGF1, FGF11, FGF9, FGF13, TGF*β*, retinoids, and Gas1 genes that are fundamental genes involved in the diversification and stabilization of the two major visual domains (RPE and NR) (Gamm et al., 2019) were mostly affected (Figure 5B). The VSX2 gene is identified as having a critical role in the maintenance of neural retina (NR) fate during early retinogenesis (Grigoryan, 2022; Gamm et al., 2019). Interestingly, the developmental homeobox genes that are hallmarks of the eye field in early development (CDH1, Wnt2, BMP7, Pitx1, Pitx2) (Gamm et al., 2019; Berber et al., 2022) were all significantly downregulated (Figures 5B,E) suggesting a direct involvement of VSX2 gene for promoting the neural developmental by repressing the early retinal developmental pathways. Fourteen genes that are associated with RPE development and functionality (Figure 5D) including the key RPE functionality gene, the RPE65 expression was significantly downregulated in EF ROs (BP-1). Importantly, RPE-specific genes such as TTR and MFSD2A that regulate the RPE functionality in the embryonic eye (Gupta et al., 2023; Wong et al., 2016), were also downregulated (BP-2) suggesting a role for VSX2 for its repressive activity in RPE differentiation (Kruczek and Swaroop, 2020).

According to Cowan et al. (2020), the temporal pattern in the appearance of neural retinal cells is RGCs, photoreceptor precursors, horizontal cells, amacrine cells, bipolar cells, and Müller cells. All the above neuronal development pathways displayed enhanced activities in EF ROs based on DEG analysis, Gen ontology (GO) analysis, and Ingenuity Pathway Analysis (IPA). This transcriptomics data was supported by immunostaining and qPCR assays. In ROs, at around D80–D120, early progenitors of cones and rods start to emerge (Afanasyeva et al., 2021; Bellapianta et al., 2022) under the influence of a set of signaling pathways comprising of Wnt, transforming growth factor beta (TGF-β), bone morphogenic proteins (BMPs), and fibroblast growth factor (FGF) (Bell et al., 2020). In addition, Visual System Homeobox genes (VSX1 and VSX2) play a crucial role in the early differentiation of photoreceptors (Afanasyeva et al., 2021; Bellapianta et al., 2022). All the above pathways exhibited differential expression in the ROs after a single exposure to EF (Figure 4), suggesting the contribution of Wnt, TGF-β, and BMP signaling pathways to the various changes observed in the EF ROs.

Based on RNA sequencing data, SAG (S-antigen visual arrestin), a major photoreceptor marker gene, exhibited significantly higher expression in both BP-1 and BP-2. Interestingly, most of the other upregulated photoreceptor-related genes belonged to the rod photoreceptor pathway, a trend that was more pronounced in the BP-1 group. RNA sequencing assay (log^2^ Fold Change 1.43, *p* < 07, DESeq), Gene Ontology analysis, and QIAGEN Ingenuity^®^ Pathway Analysis (QIAGEN IPA) also supported the above observation (Figures 4, 5). Our study that demonstrates upregulation of genes specific for rod photoreceptors suggests potential application of EF to enhance PR differentiation in ROs.

The RGC gene suppression effects on RO development caused by EF were substantiated by qPCR and immunostaining data. Several key genes including TBR1 gene which is directly involved in RGC maintenance in the retina (Liu et al., 2018) showed downregulation. Although RGC development in ROs generally declines after D80, the increased downregulation of genes related to RGC development in EF ROs suggests that extrinsic electrical fields can influence RGC development in ROs.

Increase in neuronal development and maturation in EF ROs was evidenced by the upregulation of genes and pathways associated with bipolar cell development, maturation, and functionality (GRIK1, VSX1, VSX2). Sema5B gene expression was significantly upregulated in both EF RO groups (BP-1 and BP-2). Sema5B expressed in the outer neuroblastic layer provides repulsive guidance signals to extending neurites from amacrine cell and RGC subtypes, the guidance events that are critical for retinal neural circuit formation (Matsuoka et al., 2011). GRIK1 is an established marker for bipolar cells and TFAP2A is a marker for amacrine cells (Cowan et al., 2020) both were upregulated in BP-1 and BP-2. Upregulation of genes directly associated with amacrine cell development (TFAP2A and TFAP2B) was also observed in EF ROs. The ISL1 gene that has been implicated a role in the development of on-bipolar cells and cholinergic amacrine cells (Galli-Resta et al., 1997; Elshatory et al., 2007) also showed increased expression in EF ROs. Most gene expression patterns linked to non-photoreceptor neuronal development were more similar between the BP-1 and BP-2 groups, indicating that EF likely has a broad impact on enhancing neuronal differentiation.

Müller glia in the mammalian eye exhibit neurogenic potential, with stem cell/progenitor characteristics thereby it give rise to neuronal populations of the retina (Eastlake et al., 2021). The Müller cell development originate along with retinal neurons from retinal progenitor cells (RPCs) (Ning et al., 2022). In EF ROs, the core area was more densely packed with cells that were predominantly Müller cells and photoreceptors (Figure 2). Apparent changes in the arrangement of cells in the core region of the EF-stimulated ROs can be considered as the reflection of increased differentiation of the above RO cell types. The upregulation of Müller cell development in EF ROs is demonstrated by immunostaining (Figure 2A) and RNA expression assays (Figure 5E). JAG1, a multipotent RPC marker representing gliogenesis (Mao et al., 2019; Berber et al., 2022) was also upregulated in EF ROs. Müller glial specific genes such as aquaporin 4 (Aqp4) and VSX2 (Roesch et al., 2008) were also highly expressed in the EF ROs. In addition, VIM, SLC1A3, ADM (BP-1 only), and GPR37 (BP-1 only) the other major Müller cell-related genes (Roesch et al., 2008; Ning et al., 2022), were significantly upregulated, suggesting a direct involvement of EF in increased Müller cell differentiation. Data obtained from this study suggest potential applications of EF in enhancing Müller cell populations for cell harvesting and therapeutic testing.

Present study demonstrated that EF can be a useful approach to modulate RO differentiation and maturation. Unlike using chemical molecules, the EF can be a more desirable approach for controlled enrichment of specific retinal organoid cell types. Since ROs are considered as an excellent source for photoreceptors (Lin et al., 2020; Nair et al., 2021; Thomas et al., 2021; Xue et al., 2021; Nair and Thomas, 2022a, 2022b), the enrichment of photoreceptors and synapsis can be beneficial for replacement therapies. Our study demonstrated that EF exposure can increase the population of photoreceptors and other retinal neuronal cell types in ROs by EF. While this study presents promising findings on the impact of extrinsic electric fields on retinal organoid development, further investigation is needed to examine the long-term effects of electrical stimulation in the maturation of different cell types of ROs and ensure the safety and efficacy of this approach. Investigators can fine-tune the various EF parameters and exposure time based on the degree of stimulation and the age of the ROs to determine the pathways that need to have interfered culminating in the enrichment of specific cell types that can provide the opportunity for researchers to harvest cells for conducting *in vivo* and *in vitro* assays. In summary, information obtained from this study suggests that several existing limitations in RO technology can be overcome by using suitable EF parameters without applying chemicals and small molecules.

## Data Availability

The original contributions presented in the study are publicly available. This data can be found here: https://zenodo.org/records/14226277.

## Footnotes

^1^ https://imagej.Nih.gov/ij/
