# Supplementary material for: Extrinsic electric field modulates neuronal development and increases photoreceptor population in retinal organoids
Source: Front Neurosci. 2024 Nov 29;18:1438903. doi: 10.3389/fnins.2024.1438903 (PMC11639233; doi:10.3389/fnins.2024.1438903)
Supplement: Supplementary file 1 [file Data_Sheet_1.docx]

**
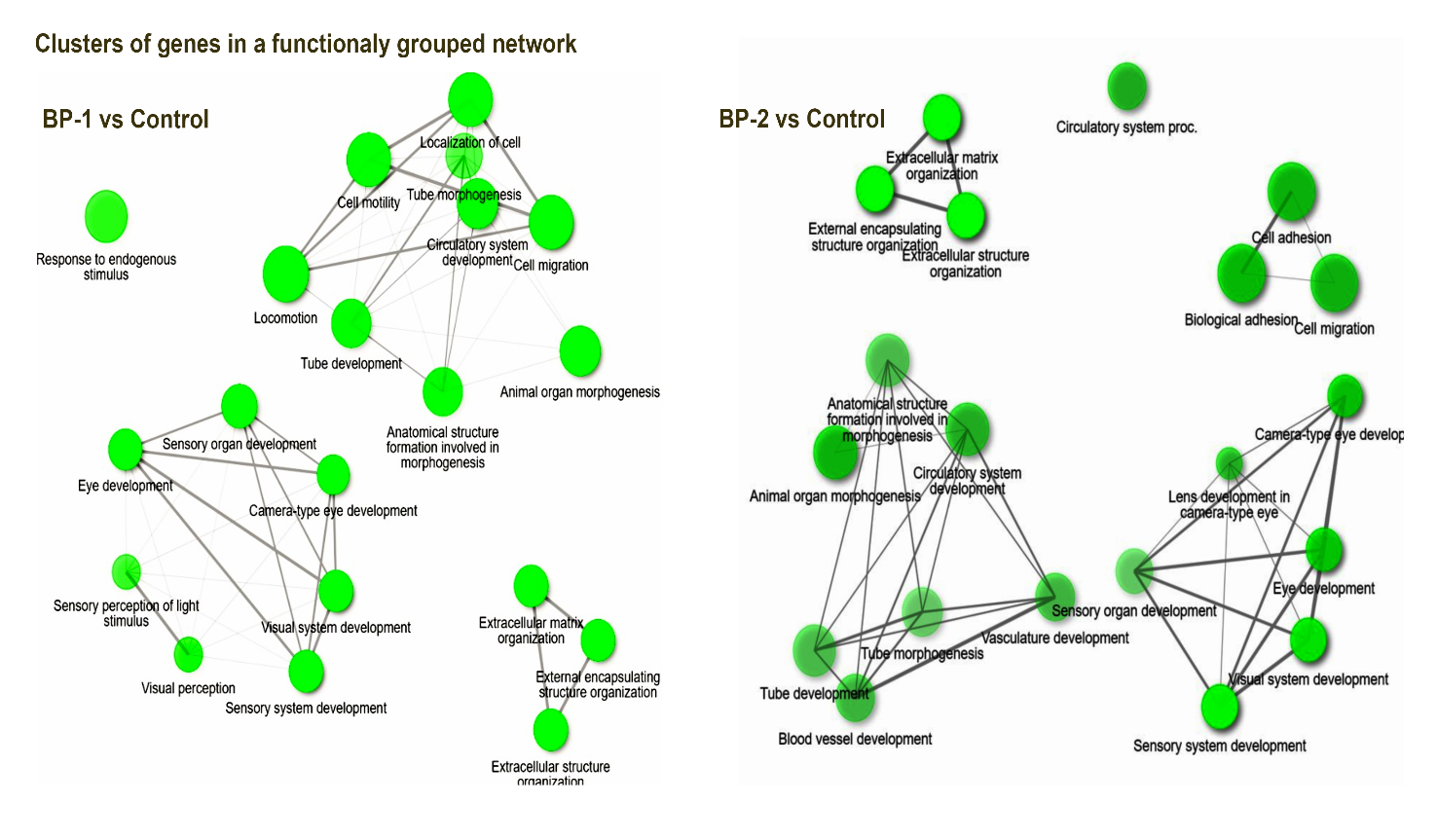
Supplementary Figure 1. Gene ontology analysis showing clusters of genes in a functionally grouped network**. Top 10 GO biological category annotations for the differentially expressed genes (DEGs) identified based on the RNA-seq data for control vs electrically stimulated retinal organoid samples (a. BP-1 vs. control, b. BP-2 vs control). A term with P < 0.05 that is considered to be significantly overexpressed is included in this cluster.


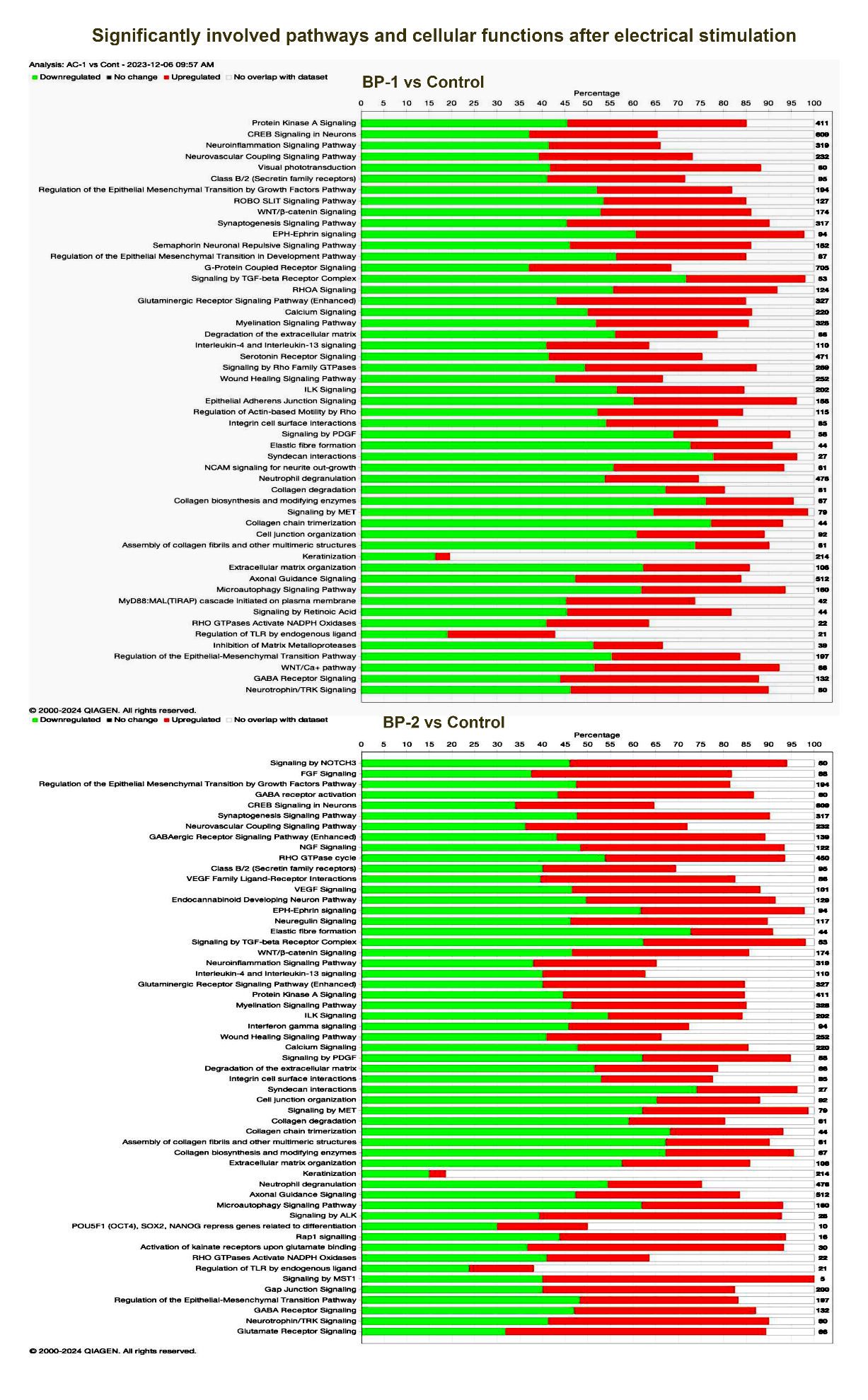


**Supplementary Figure 2.** **Ingenuity Pathway Analysis (IPA) data**. IPA of differentially expressed genes in retinal organoids showed as a graphical summary representing networks of the major pathways identified as the most significant in the differential transcriptomics data (P < 0.05). The degree of gene downregulation (green) and upregulation (red) are denoted by color codes and the total number of genes belonging to each category is given in the right panel.


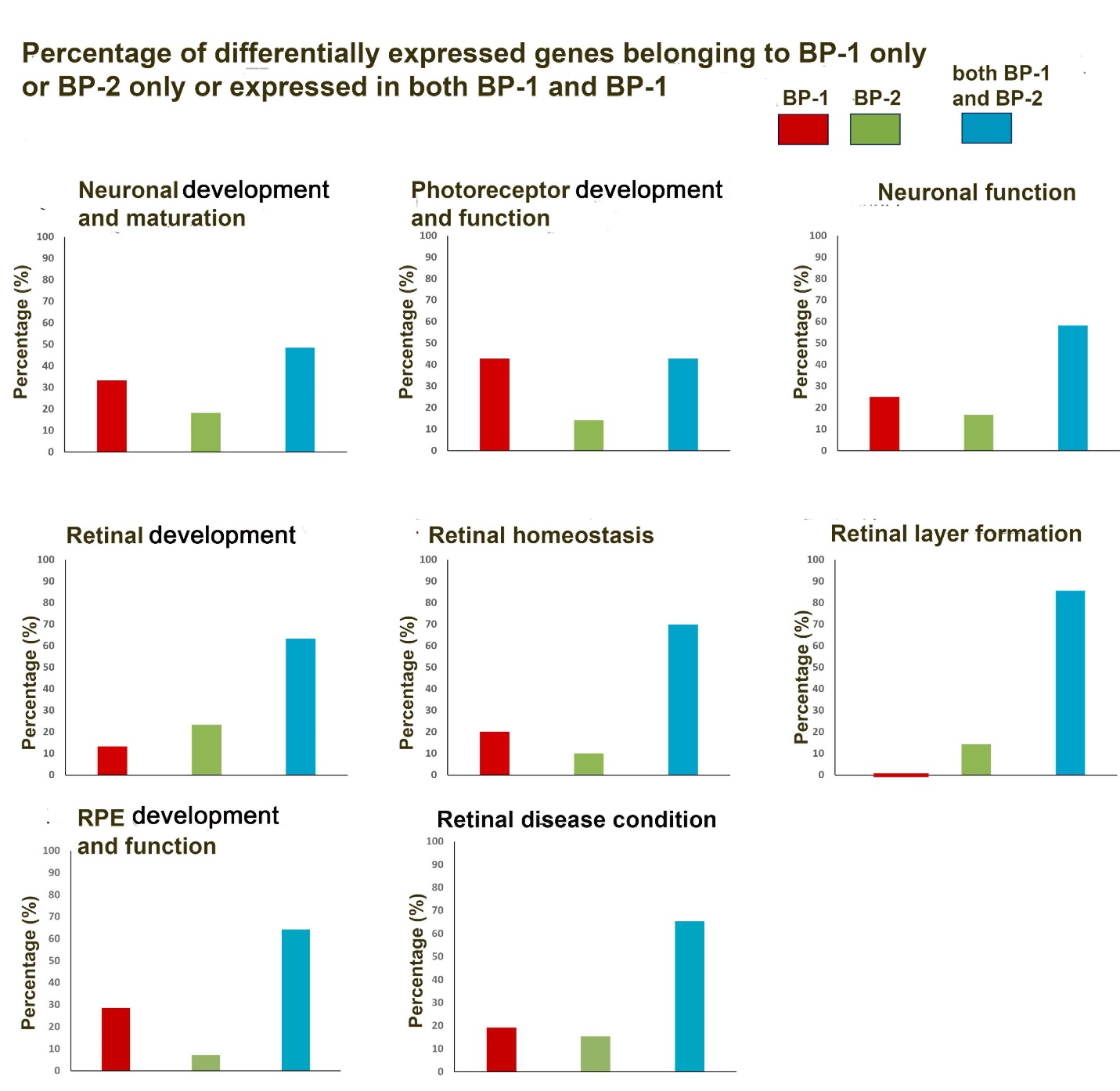


**Supplementary Figure 3. Number of upregulated and downregulated genes belonging to the key retinal developmental pathways**. The bar diagram shows the percentage of differentially expressed genes in BP-1 and BP-2 retinal organoid groups.

**Table 1** **List of the genes with its corresponding catalog numbers**

| Probe List | Gene Alias | Cat#(Thermo Fisher) |
| --- | --- | --- |
| Recoverin | Rcvrn | Hs00610056_m1 |
| Red blue Opsin | OPN1SW | Hs00181790_m1 |
| CRX | CRX | Hs00230899_m1 |
| vGLUT1 | SLC17A7 | Hs00220404_m1 |
| GAPDH | GAPDH | Hs00266705_g1 |
| SAG | S-antigen visual arrestin | Hs01027187_m1 |
| Ki67 | MKI67 | Hs01032443_m1 |

**Table 2 List of Antibodies Used**

**Primary antibodies**

| **Antigen** | **Species** | **Specific for** | **Dilution** | **Supplier** | **Catalog #** |
| --- | --- | --- | --- | --- | --- |
| Recoverin | Rabbit | Photoreceptors, cone bipolar cells | 1:2K | Millipore | AB5585-I |
| Vimentin | Rabbit | Muller cells | 1:300 | Abcam | ab1373 |
| CRX | Rabbit | Photoreceptor progenitor | 1:300 | Santa Cruz Biotechnology | sc-30150 |
| Synaptophysin | Goat | Membrane protein of synaptic vesicles | 1:300 | Novus Biologicals | AF5555 |

**Secondary antibodies**

| **Conjugate** | **Species** | **Specific for** | **Dilution** | **Supplier** | **Catalog #** | **RRID** |
| --- | --- | --- | --- | --- | --- | --- |
| Alexa Fluor 488 | Donkey | Rabbit IgG (H + L) | 1:400 | Jackson ImmunoResearch (West Grove, PA, United States) | 711-545-152 | AB_2313584 |
| Rhodamine Red-X | Donkey | Rabbit IgG (H + L) | 1:400 | Jackson ImmunoResearch | 711-295-152 | AB_2340613 |

**Table 3 List of Differentially Expressed Genes that were Upregulated and Downregulated**

1. **Neuronal Maintenance and Functionality BP-1**

BP-1

**b. Neuronal Maintenance and Functionality BP-2**

BP-2

**c. Neuronal Development and Maturation BP-1**

**d. Neuronal Developmnent and Maturation BP-2**

**BP-2**

**f. Photoreceptor Development and Function BP-2**

BP-2

**e. Photoreceptor Development and Function BP-1**

BP-1

**h. Retinal Development and Function BP-2**

**g. Retinal Development and Function BP-1**

**n. RPE Development and Function BP-2**

**m. RPE Development and Function BP-1**
